# Reproducibility: let’s get it right from the start

**DOI:** 10.1038/s41467-018-06012-8

**Published:** 2018-09-12

## Abstract

From September 12th 2018, *Nature Communications* will be setting a higher standard of data reporting for papers under peer review. We believe that sharing raw data at an early stage with editors and reviewers is the best way to build confidence in the reproducibility of your findings. Learn here how to ensure that your paper makes the grade.

Whether you prefer to call it a crisis, a challenge or a revolution, the growing awareness of reproducibility as an important issue in science is surely a cause for optimism. In biomedical research in particular, journals now compete with each other to demonstrate the strength of their reproducibility credentials, and play an increasingly proactive role in setting standards for how data and methods should be reported in their pages. Many journals and funding bodies have developed checklists and guidelines with the aim of ensuring that published papers clearly demonstrate that the findings were reproducible, and provide sufficient information for readers to reproduce the work for themselves.

At *Nature Communications*, we have typically asked our life science authors to complete such a checklist when they resubmit a revised version of their manuscript in response to reviewer comments. We have also deployed subject-specific checklists to set standards for the reporting of magnetic resonance imaging, ChIP-seq, laser and solar cell experiments. However, with the exception of the laser and solar cells checklists, they have generally not been made available to peer reviewers and have been implemented primarily as a guidance document for authors rather than a list of strict requirements.

Despite this guidance, our editors have on occasion identified serious problems with papers after having requested more detailed information, and this can occur at a relatively late stage in review, resulting in a waste of time for reviewers and authors alike. Ensuring that reporting is transparent, right from the start of the peer review process, would allow our reviewers to scrutinize the level of support for the findings with greater confidence and at a point where any problems can more easily be resolved.

For this reason, *Nature Communications* is now joining the other Nature journals in requiring authors to complete all relevant checklists at the point their paper is sent for peer review. In the life sciences, we will require the reporting summary to be completed, which incorporates all of the life science-related checklists into a single document. In the physical sciences, the solar cells and lasing reporting summaries will continue to be mandated at this stage. As these reporting summaries are already in use by editors at *Nature* and the Nature Research journals, authors transferring peer-reviewed papers from these journals to *Nature Communications* will not have to complete additional checklists, and in common with these journals, the reporting summary will be made available to reviewers in every round of peer review and published alongside the article. Publishing these reporting summaries will establish them as a key part of the paper, allowing readers to verify and build on the study.

 “…the trio of published checklists, source data and reviewer reports together will represent powerful proof of the strength and rigor of the published work and the peer review process that helped to improve it.”

To further promote transparency, we will also be asking our authors to supply for publication the source data underlying any graphs and charts, and uncropped, unprocessed scans of any blots or gels. In order to avoid delaying the first round of peer review, we will initially only request that source data be supplied with the revised versions of the manuscript, unless we feel that it would be particularly important for the reviewers to have access to these data at an earlier stage. However, we strongly encourage authors to lead the way in promoting transparency by making sure that their work fulfils or exceeds our criteria right from the start. Here are four simple steps you can take to give your paper the best possible chance of flying through peer review:

1. **Define your** ***n*** **numbers**: just stating ‘*n* = 5’ is no longer sufficient—it must be clear what your *n* number refers to for each experiment. For example, did you do the experiment on five plants, on five tissue samples from the same plant, or did you run the same sample for analysis five times over? You should be able to justify your choice of sample size and statistical tests in the reporting summary.
2. **Show us the spread**: bar charts with error bars are commonly used to depict numerical data, but they can be misleading when sample sizes are small. Wherever possible, use graph types that include individual data points to directly show the spread of the data.
3. **Make it available**: before submitting your paper, take a close look at our policies on the availability of materials, data and code, and craft clear availability statements in your paper that explain to reviewers and future readers how they can access everything they might need to reproduce your findings. For some types of data, deposition in an approved publicly accessible database is mandatory for publication, but don’t stop there: include source data with your manuscript, and make use of repositories such as figshare to make other data available to your reviewers and readers. For datasets with high re-use value, consider also submitting a data descriptor to our sister journal, Scientific Data, and making the dataset publicly available through one of their recommended repositories.
4. **Let us be your guide**: the reporting summary and solar cells and lasing reporting summaries detail a lot of important information that can be used not just to help you describe your experimental design, figures of merit, the materials used and the analysis employed, but also to plan the experiment in the first place. Taking care to consider these requirements before you conduct your experiments will improve your chances of successful publication in any journal.

Finally, with the best will in the world, making source data available to peer reviewers does not necessarily mean that they will be able to scrutinise and reanalyse every aspect of it in detail; this would in some cases be a mammoth task. However, we hope that our reviewers will make use of this resource and freely comment on any issues in their reports. We also hope that our authors will choose to publish these reviewer comments alongside their articles by opting into our transparent peer review scheme, as the trio of published checklists, source data and reviewer reports together will represent powerful proof of the strength and rigor of the published work and the peer review process that helped to improve it.

